# Real-world data of long-term survival in patients with T-cell lymphoma who underwent stem cell transplantation

**DOI:** 10.1038/s41408-023-00868-w

**Published:** 2023-06-26

**Authors:** Dong Won Baek, Joon Ho Moon, Jae Hoon Lee, Ka-Won Kang, Ho Sup Lee, Hyeon-Seok Eom, Enuyoung Lee, Ji Hyun Lee, Jeong-Ok Lee, Seong Kyu Park, Seok Jin Kim, Keon Hee Yoo, Sung-Soo Yoon, Youngil Koh, Hyoung Jin Kang, Jong-Ho Won, Chuhl Joo Lyu, Seung Min Hahn, Jung-Hee Lee, Joon Seong Park, Jae-Cheol Jo, Yeung-Chul Mun, Deok-Hwan Yang, Ga-Young Song, Sung-Nam Lim, Sang Kyun Sohn, The Korean Society of Blood and Marrow Transplantation

**Affiliations:** 1 Department of Hematology/Oncology, Kyungpook National University Hospital, School of Medicine, Kyungpook National University, Daegu, South Korea; 2 Hematology, Gachon University Gil Medical Center, Incheon, South Korea; 3 Division of Hematology-Oncology, Department of Internal Medicine, Korea University College of Medicine, Seoul, South Korea; 4 Division of Hematology, Department of Internal Medicine, Kosin University College of Medicine, Kosin University Gospel Hospital, Busan, South Korea; 5 Center for Hematologic Malignancy, National Cancer Center, Seoul, South Korea; 6 Division of Hematology-Oncology, Department of Internal Medicine, Dong-A University College of Medicine, Busan, South Korea; 7 Department of Internal Medicine, Seoul National University Bundang Hospital, Seoul National University College of Medicine, Seoul, South Korea; 8 Division of Hematology-Oncology, Department of Internal Medicine, Soonchunhyang University Bucheon Hospital, Bucheon, South Korea; 9 Division of Hematology-Oncology, Department of Medicine, Samsung Medical Center, Sungkyunkwan University School of Medicine, Seoul, South Korea; 10 Department of Pediatrics, Samsung Medical Center, Sungkyunkwan University School of Medicine, Seoul, South Korea; 11 Department of Internal Medicine, Seoul National University Hospital, Seoul, South Korea; 12 Department of Pediatrics, Seoul National University College of Medicine, Seoul National University Cancer Research Institute, Seoul National University Children’s Hospital, Seoul, South Korea; 13 Division of Hematology-Oncology, Department of Internal Medicine, Soonchunhyang University Seoul Hospital, Seoul, South Korea; 14 Department of Pediatric Hematology Oncology, Severance Hospital, Yonsei University, Seoul, South Korea; 15 Department of Hematology, Asan Medical Center, University of Ulsan College of Medicine, Seoul, South Korea; 16 Department of Hematology-Oncology, Ajou University School of Medicine, Suwon, South Korea; 17 Department of Hematology and Oncology, Ulsan University Hospital, University of Ulsan College of Medicine, Ulsan, South Korea; 18 Department of Internal Medicine, Ewha Womans University School of Medicine, Seoul, South Korea; 19 Department of Hematology-Oncology, Chonnam National University Hwasun Hospital, Chonnam National University Medical School, Hwasun-gun, Jeollanam-do South Korea; 20 Division of Hematology-Oncology, Department of Internal Medicine, Inje University College of Medicine, Haeundae Paik Hospital, Busan, South Korea

**Keywords:** T-cell lymphoma, Cancer immunotherapy

## Abstract

This study aimed to identify the benefits of autologous-stem cell transplantation (auto-SCT) and allogeneic-SCT (allo-SCT) in patients with aggressive T-cell lymphomas to aid in the selection of transplantation type in clinical practice. This study retrospectively analyzed data from 598 patients who underwent transplantation for T-cell lymphomas from 2010 to 2020. In total, 317 patients underwent up-front SCT as consolidation therapy. The 3-year progression-free survival (PFS) and overall survival (OS) were 68.7% and 76.1%, respectively. Patients who underwent auto-SCT had significantly better OS (*p* = 0.026) than those who underwent allo-SCT; however, no statistical difference in PFS was found. Transplantation was used as a salvage therapy in 188 patients who had relapsed/refractory disease. Overall, 96 (51.1%) patients underwent auto-SCT and 92 (48.9%) patients underwent allo-SCT. Auto-SCT improved long-term survival in patients with complete remission (CR). Allo-SCT demonstrated better 3-year PFS in patients with partial remission and relapsed/refractory disease status. However, >50% of patients died within 1 year of allo-SCT. As a consolidative therapy, up-front auto-SCT demonstrated a survival benefit. Auto-SCT was also effective in patients who achieved CR after salvage therapy. If the disease persists or cannot be controlled, allo-SCT may be considered with reduced intensity conditioning.

## Introduction

Stem cell transplantation (SCT) has been regarded as an important strategy for providing a complete cure in the treatment of aggressive peripheral T-cell lymphomas (PTCLs) [1–4]. Previous studies have shown that consolidative up-front high-dose chemotherapy followed by autologous-SCT (auto-SCT) reduces relapse rate and improves long-term outcomes [5, 6]. Allogeneic-SCT (allo-SCT) may be an effective option for patients with aggressive diseases through conditioning chemo- or chemoradiotherapy and graft-versus-lymphoma (GVL) effect [7, 8]. Auto-SCT might provide survival benefits in patients with relapsed/refractory status. However, some previous studies have suggested that allo-SCT is more beneficial for patients with chemo-resistant or relapsed status [9, 10]. According to a recent meta-analysis, auto-SCT is the most commonly used modality for chemosensitive patients, whereas allo-SCT has been used as a salvage approach [11]. However, the roles of auto- or allo-SCT remain controversial, owing to the negative effects of treatment-related toxicity.

Most studies on transplantation have been retrospective in nature, and there are few large-scale prospective studies. Furthermore, there may be a patient selection bias wherein transplant-eligible patients in good condition have a better prognosis. However, for clinicians, preventing disease progression is most important when patients exhibit a specific response to front-line or salvage chemotherapy; therefore, SCT is considered in such cases. Proper stem cell collection is essential for auto-SCT, and finding a suitable donor and reducing treatment-related mortality (TRM) are major hurdles to successful allo-SCT. Because of these factors, there has been no clear guideline on whether to proceed with transplantation or which transplant method should be selected between auto- and allo-SCT.

In this study, we retrospectively analyzed long-term survival outcomes in patients with T-cell lymphomas who underwent auto- or allo-SCT as a consolidation or salvage therapy. The benefits of auto- and allo-SCT were also compared to aid in the selection of transplantation type in clinical practice.

## Methods

### Patients

Data from 598 patients who underwent transplantation for T-cell lymphomas from 2010 to 2020 were retrospectively analyzed using the Korean Society of Blood and Marrow Transplantation (KSBMT) registry. Patients aged ≥15 years who had been treated for biopsy-confirmed PTCLs according to the 2008 World Health Organization (WHO) classification were included in this study [12]. PTCLs included peripheral T-cell lymphoma not otherwise specified (PTCL-NOS), angioimmunoblastic T-cell lymphoma (AITL), anaplastic large cell lymphoma (ALCL), extranodal NK/T-cell lymphoma, nasal type, and enteropathy-associated T-cell lymphoma. Most patients received CHOP-based chemotherapy as a first-line treatment, and there was no institutional policy to determine SCT. Chemotherapy and conditioning regimens before transplantation were selected by local physicians at each center. The treatment response to chemotherapy and SCT was assessed based on the International Workshop Criteria [13]. A complete remission (CR) is defined as the complete disappearance of all detectable clinical evidence of disease, whereas a partial remission (PR) is defined as a regression of measurable disease with no new sites. Refractory status is defined as no response to prior treatment at all, and relapsed status is defined as any new lesion or an increase in the number of previously involved sites from nadir by ≥50%.

Patient clinical data were collected from the KSBMT registry. This study was approved by the Institutional Review Board of Kyungpook National University Hospital and was conducted in accordance with the Declaration of Helsinki.

### Statistical analysis

This retrospective study aimed to identify the survival benefits of transplantation in patients with specific treatment statuses. Furthermore, the benefits of auto- and allo-SCT were compared to aid in the selection of transplantation type in clinical practice. Progression-free survival (PFS) was calculated as the time from transplantation to disease progression, relapse, or death from any cause. Overall survival (OS) was calculated as the time from transplantation to death from any cause or the last follow-up. The probabilities of PFS and OS were calculated using the Kaplan–Meier method and compared using the log-rank test. The Cox regression model was used to identify the factors that affect long-term survival including patient- and disease-related variables (age at the time of SCT, sex, previous SCT history, and response to previous chemotherapy). Transplantation-related factors were also included such as the type of SCT (auto-SCT vs. allo-SCT), intensity of the conditioning regimen, and donors for allo-SCT. Factors with a *p*-value of ≤0.1 in the univariate analysis were retained in the multivariate analysis. The stepwise method for variable selection was used. For each factor, the hazard ratio and 95% confidence interval were calculated. A *p*-value of <0.05 was considered to indicate statistical significance. Propensity score-based matching approaches was also used to reduce differences in the characteristics between the auto- and allo-SCT groups. Confounding factors such as age at the time of SCT, sex, disease subtype, and disease status were applied for matching. Statistical analyses were conducted using R statistical software 3.6.2 (the R Foundation for Statistical Computing, Vienna, Austria, available at http://www.r-project.org).

## Results

### SCT as a part of first-line therapy

In total, 317 patients underwent upfront autologous (*n* = 254) or allogeneic (*n* = 63) SCT as consolidation therapy following first-line chemotherapy. The baseline characteristics of the patients are presented in Table 1. The median age of the auto-SCT group was 51.1 (range, 15.3–68.7) years, with 130 patients (51.2%) aged >50 years, whereas the median age of the allo-SCT group was 41.9 (range, 15.1–62.9) years, with only 15 patients (23.8%) aged >50 years. According to the WHO classification, 107 (42.1%) patients had PTCL-NOS, 54 (21.3%) had extranodal NK/T-cell lymphoma, 48 (18.9%) had AITL, and 28 (11.0%) had ALK-negative ALCL in the auto-SCT group. In the allo-SCT group, most patients were treated for PTCL-NOS (*n* = 34, 54.0%) and extranodal NK/T-cell lymphoma (*n* = 20, 31.7%). The majority of the patients in both groups underwent transplantation after achieving CR. However, the proportion of patients with PR was higher in the allo-SCT group.

**Table 1:** Baseline characteristics of patients who underwent up-front stem cell transplantation as a maintenance therapy (*n* = 317).

Characteristics	Autologous SCT	Allogeneic SCT	*p*-value
	No. (%)	No. (%)	
No. of patients	254 (80.1)	63 (19.9)	
*Age at the time of SCT, years*			0.013
Median (range)	51.1 (15.3–68.7)	41.9 (15.1–62.9)	
≤20	16 (6.3)	14 (22.2)	
21–30	16 (6.3)	8 (12.7)	
31–40	26 (10.2)	7 (11.1)	
41–50	66 (26.0)	19 (30.2)	
51–60	86 (33.9)	11 (17.5)	
> 60	44 (17.3)	4 (6.3)	
*Sex*			0.547
Male	173 (68.1)	46 (73.0)	
Female	81 (31.9)	17 (27.0)	
*Ann Arbor stage at diagnosis*			0.918
III, IV	242 (95.3)	62 (98.4)	
*Histopathology*			0.010
PTCL-NOS	107 (42.1)	34 (54.0)	
Extranodal NK/T-cell lymphoma	54 (21.3)	20 (31.7)	
AITL	48 (18.9)	4 (6.3)	
ALCL, ALK (−)	28 (11.0)	4 (6.3)	
EATL	9 (3.5)	0 (0)	
Others	8 (3.1)	1 (1.6)	
*First-line chemotherapy*			0.157
CHOP(E)^a^	191 (75.2)	39 (61.9)	
Others	63 (24.8)	24 (38.1)	
*Disease status at the time of SCT*			0.109
CR	190 (74.8)	38 (60.3)	
PR	64 (25.2)	25 (39.7)	

*AITL* angioimmunoblastic T-cell lymphoma, *ALCL* anaplastic large-cell lymphoma, *ALK* anaplastic lymphoma kinase, *CR* complete remission, *EATL* enteropathy-associated T-cell lymphoma, *PR* partial remission, *PTCL-NOS* peripheral T-cell lymphoma not otherwise specified, *SCT* stem cell transplantation.

^a^CHOP(E), cyclophosphamide, doxorubicin, vincristine, and prednisone (etoposide).

**Fig. 1:**
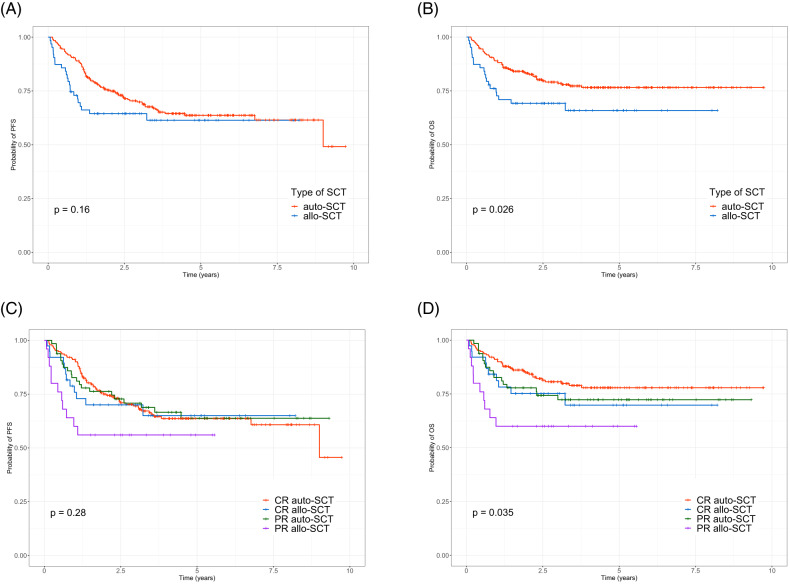
Kaplan–Meier curves. No significant difference in progression-free survival was found between the auto- and allo-SCT groups (**A**). However, patients who underwent auto-SCT exhibited better overall survival (**B**) than patients with allo-SCT did. Progression-free survival showed no statistical significance with SCT type and disease status at the time of transplantation (**C**). However, patients with CR and underwent auto-SCT had superior long-term survival (**D**).

The median follow-up duration of all patients was 5.5 (range, 0.1–10.3) years. In total, 53 (16.7%) patients had disease relapse or progression after SCT, and 74 patients (23.3%) died. The 3-year PFS and OS were 68.7%, and 76.1% respectively. Survival curves of PFS and OS are depicted in Supplementary Fig. 1. No significant difference in PFS was found between patients with CR and PR. However, patients who achieved CR before SCT showed a trend of better OS (*p* = 0.059) than those with PR (Supplementary Fig. 2). In the survival analysis including both patients with CR and PR, the auto-SCT group showed significantly better OS (*p* = 0.026) than the allo-SCT group, although no statistical difference in PFS was noted (Fig. 1A, B). The Kaplan–Meier curves revealed that patients with CR and underwent auto-SCT had significantly better long-term survival than those with PR or who underwent allo-SCT (Fig. 1C, D). Supplementary Table 1 summarizes the survival data according to the patient’s status. In patients with CR and PR, the allo-SCT group had a lower relapse rate than the auto-SCT group. However, the non-relapse mortality (NRM) of the allo-SCT group was higher than that of the auto-SCT group among patients with CR and PR (Supplementary Fig. 3A–D). During and after upfront transplantation, 54 patients in the auto-SCT group and 20 patients in the allo-SCT group died (Supplementary Table 2). In particular, the 1-year mortality after SCT was much higher in the allo-SCT group than in the auto-SCT group. Moreover, all deaths (*n* = 10) of patients with PR who underwent allo-SCT occurred within the first year of transplantation (Supplementary Table 1).

Patients aged <50 years had significantly better long-term survival than older patients. In patients aged <50 years, auto- and allo-SCT showed no significant difference in survival curves. However, in patients aged ≥50 years, auto-SCT resulted in better OS than allo-SCT (*p* = 0.004) (Supplementary Fig. 4A–F). In the subgroup analysis, patients with PTCL-NOS who underwent auto-SCT showed better survival benefits (PFS, *p* = 0.037; OS, *p* = 0.017) than those who underwent allo-SCT, whereas other subtypes showed statistically similar long-term survival between transplant types. In the multivariate analysis, age of <50 years at the time of SCT and auto-SCT were favorable factors for PFS and OS (Supplementary Table 3).

In the propensity score matching analysis applying age, sex, disease status, disease subtype, and type of transplantation, 59 patients with CR/PR in each auto- and allo-SCT group were matched and analyzed. The matching results were added as a histogram in the supplementary materials. In the propensity score-matched dataset, no significant difference in PFS was found between the auto- and allo-SCT groups. The auto-SCT group showed a trend of better OS (*p* = 0.087) than the allo-SCT group (Supplementary Fig. 5A–C).

### SCT for patients with relapsed/refractory disease

SCT was used as a salvage therapy in 188 patients who had relapsed or refractory disease following intensive chemotherapy. Overall, 96 (51.1%) patients underwent auto-SCT, whereas 92 (48.9%) patients underwent allo-SCT. The clinical characteristics of the patients are summarized in Table 2. At the time of transplantation, the median age of the auto-SCT and allo-SCT groups were 47.6 (range, 15.2–69.4) and 43.8 (15.1–70.9) years, respectively. Most patients in both groups underwent transplantation for PTCL-NOS, extranodal NK/T-cell lymphoma, AITL, and ALK-negative ALCL. Moreover, 55 (57.3%) patients in the auto-SCT group and 51 (55.5) in the allo-SCT group had undergone transplantation in the relapsed/refractory disease status.

**Table 2:** Baseline characteristics of patients who underwent stem cell transplantation as a part of salvage therapy (*n* = 188).

Characteristics	Autologous SCT	Allogeneic SCT	*p*-value
	No. (%)	No. (%)	
No. of patients	96 (51.1)	92 (48.9)	
*Age at the time of SCT, years*			0.528
Median (range)	47.6 (15.2–69.4)	43.8 (15.1–70.9)	
≤20	10 (10.4)	13 (14.1)	
21–30	10 (10.4)	11 (12.0)	
31–40	13 (13.5)	11 (12.0)	
41–50	26 (27.1)	18 (19.6)	
51–60	25 (26.0)	30 (32.6)	
>60	12 (12.5)	9 (9.8)	
*Sex*			0.757
Male	68 (70.8)	68 (73.9)	
Female	28 (29.2)	24 (26.1)	
*Histopathology*			0.185
PTCL-NOS	32 (33.3)	39 (42.4)	
Extranodal NK/T-cell lymphoma	22 (22.9)	15 (16.3)	
AITL	17 (17.7)	20 (21.7)	
ALCL, ALK (−)	13 (13.5)	13 (14.1)	
ALCL, ALK (+)	6 (6.3)	2 (2.2)	
EATL	4 (4.2)	2 (2.2)	
Others	2 (2.1)	1 (1.1)	
*Disease status at the time of SCT*			0.001
CR2	27 (28.1)	30 (32.6)	
>CR2	5 (5.2)	3 (3.3)	
≥PR2	9 (9.4)	8 (8.7)	
Refractory	47 (49.0)	18 (19.6)	
Relapsed	8 (8.3)	33 (35.9)	
*No. of SCT*			0.072
1	88 (91.7)	75 (81.5)	
2	7 (7.3)	15 (16.3)	
≥3	1 (1.0)	2 (2.2)	

*AITL* angioimmunoblastic T-cell lymphoma, *ALCL* anaplastic large-cell lymphoma; *ALK* anaplastic lymphoma kinase, *CR* complete remission, *EATL* enteropathy-associated T-cell lymphoma, *PR* partial remission, *PTCL-NOS* peripheral T-cell lymphoma not otherwise specified, *SCT* stem cell transplantation.

**Fig. 2:**
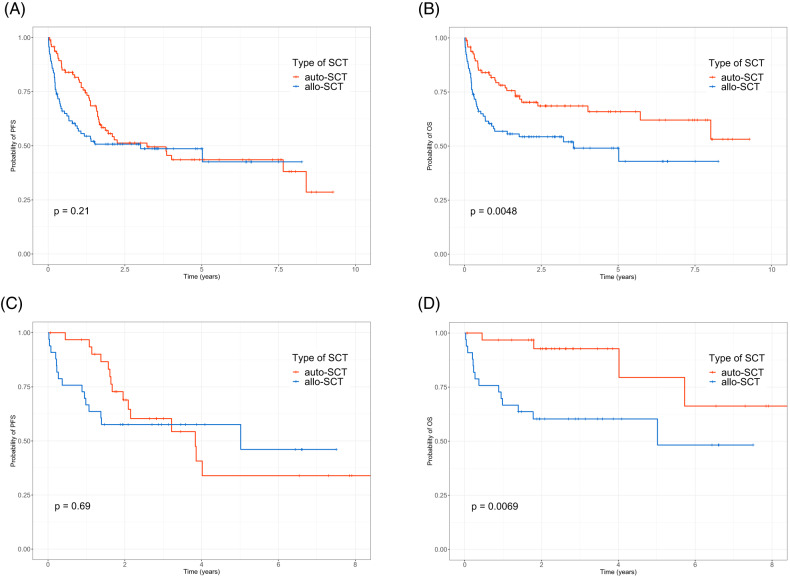
Kaplan–Meier curves. No significant difference in progression-free survival was found between the auto- and allo-SCT groups (**A**). Patients who underwent auto-SCT showed better OS than those who underwent allo-SCT (**B**). The progression-free survival showed no statistical significance with the SCT type in patients who achieved CR (**C**). However, patients with CR who underwent auto-SCT reported considerably better OS (**D**).

The median follow-up duration was 21.5 (range, 0.1–112.8) months. The estimated 3-year PFS and OS of all patients were 50.1% and 61.6%, respectively. The 3-year OS in patients who achieved CR after salvage therapy was 75.7%. The 3-year OS was 63.7% in patients with PR, 54.3% in those with refractory disease, and 47.8% in those with relapsed disease. There was no significant difference in PFS between the auto- and allo-SCT groups. Patients who underwent auto-SCT showed better OS than those who underwent allo-SCT. In particular, patients with CR who underwent auto-SCT reported considerably better OS (Fig. 2A–D). The cumulative incidence of relapse was higher in the auto-SCT group, whereas the NRM was higher in the allo-SCT group (Supplementary Fig. 6A–D). Supplementary Table 4 summarizes patient outcomes based on disease status at the time of transplantation. Auto-SCT resulted in better long-term survival in patients with CR in terms of 3-year PFS and OS. In patients with PR, refractory disease, or relapsed status, allo-SCT resulted in a better 3-year PFS. However, >50% of patients died within 1 year of allo-SCT. In patients with CR/PR, disease progression and infection were the most common cause of death in the auto-SCT and allo-SCT groups, respectively. In the auto-SCT group, most of the patients with relapsed/refractory status died from infection and disease progression. However, infection and graft-versus-host disease were major causes of death in the allo-SCT group (Supplementary Table 5).

In the multivariate analysis, the absence of a previous SCT history was a favorable factor for OS at the time of allogeneic transplantation, and chemoresistant disease was an adverse factor affecting long-term survival (Table 3). Receiving allogeneic transplantation was adverse factor for OS in patients with CR/PR status at the time of SCT (Supplementary Table 6). In patients with relapsed/refractory status at the time of SCT, a previous history of transplantation was an adverse factor, whereas having experienced a certain response to chemotherapy previously was a favorable factor affecting PFS and OS (Supplementary Table 7).

**Table 3:** Factors affecting long-term survival in patients who underwent stem cell transplantation as salvage therapy.

	Univariate			Multivariate		
	HR	95% CI	*p*-value	HR	95% CI	*p*-value
(1) *Progression-free survival*						
Age at SCT, ≥ 50 vs. < 50	1.07	0.700–1.621	0.767			
Female vs. male	0.93	0.587–1.484	0.771			
Previous SCT, no vs. yes	0.82	0.495–1.350	0.430			
Allogeneic-SCT vs. autologous-SCT	1.44	0.882–2.338	0.146			
Chemosensitivity, relapsed/refractory vs. CR/PR	1.50	0.978–2.298	0.063	1.51	0.992–2.288	0.055
(2) *Overall survival*						
Age at SCT, ≥ 50 vs. < 50	0.96	0.601–1.542	0.873			
Female vs. male	0.91	0.537–1.554	0.740			
Previous SCT, no vs. yes	0.59	0.340–1.016	0.057	0.58	0.338–0.998	0.049
Allogeneic-SCT vs. autologous-SCT	2.57	1.499–4.420	<0.001	2.61	1.528–4.442	<0.001
Chemosensitivity, relapsed/refractory vs. CR/PR	2.02	1.228–3.334	0.006	1.99	1.222–3.255	0.006

*CI* confidence interval, *CR* complete remission, *HR* hazard ratio, *PR* partial remission, *SCT* stem cell transplantation.

In the salvage setting, 92 patients underwent allo-SCT. Sixty-three patients received reduced intensity conditioning (RIC), whereas 29 patients had myeloablative conditioning (MAC) regimen. Patients who received RIC were relatively older. Supplementary Table 8 summarizes comparative data related to allo-SCT according to the conditioning intensity. In the subgroup analysis of patients who underwent allo-SCT, total body irradiation (TBI) and intensity of the conditioning regimen were factors that affect PFS and OS. According to the multivariate analysis, patients with TBI and those who received a RIC regimen had significantly better outcomes (Table 4). Donor types (HLA full-matched versus haploidentical donor; sibling donor versus unrelated donor) were not significant factors for survival.

**Table 4:** Factors affecting long-term survival in patients who underwent allogeneic stem cell transplantation as salvage therapy.

	Univariate			Multivariate		
	HR	95% CI	*p*-value	HR	95% CI	*p*-value
(1) *Progression-free survival*						
Age at SCT, ≥50 vs. <50	0.83	0.455–1.521	0.551			
Female vs. male	1.63	0.828–3.211	0.157			
No. of SCT, ≥2 vs. 1	0.79	0.422–1.483	0.465			
TBI, performed vs. none	0.49	0.256–0.921	0.027	0.42	0.221–0.808	0.009
Conditioning intensity, MAC vs. RIC	2.03	1.213–4.379	0.011	2.04	1.125–3.685	0.019
Donor, haploid vs. full-matched	1.46	0.027–2.211	0.791			
(2) *Overall survival*						
Age at SCT, ≥50 vs. <50	0.80	0.426–1.513	0.496			
Female vs. male	1.48	0.723–3.016	0.284			
No. of SCT, ≥2 vs. 1	0.72	0.377–1.386	0.328			
TBI, performed vs. none	0.45	0.226–0.890	0.022	0.37	0.180–0.735	0.004
Conditioning intensity, MAC vs. RIC	2.46	1.266–4.763	0.008	2.56	1.348–4.853	0.004
Donor, haploid vs. full-matched	2.06	0.958–4.434	0.064	1.90	0.915–3.954	0.085

*CI* confidence interval, *HR* hazard ratio, *MAC* myeloablative conditioning, *RIC* reduced-intensity conditioning, *SCT* stem cell transplantation, *TBI* total body irradiation.

Propensity score-matched approaches were also conducted in the salvage setting. Among patients with CR/PR, 36 patients each in the auto- and allo-SCT groups were matched. In patients with relapsed/refractory disease, 24 patients in each group were matched. The matching results were added as a histogram in the supplementary materials. In patients with CR/PR and relapsed/refractory disease at the time of SCT, no significant differences in PFS were found between the auto- and allo-SCT groups. However, among patients with CR/PR, the auto-SCT group showed better OS (*p* = 0.018) than the allo-SCT group (Supplementary Fig. 7A–F).

## Discussion

We evaluated the long-term outcomes of patients with T-cell lymphomas who underwent transplantation as a part of consolidation or salvage therapy using data from the KSBMT registry from 2010 to 2020. Patients who underwent consolidative auto-SCT showed better OS than those who underwent allo-SCT in the up-front SCT group. Survival outcomes in the salvage group varied depending on the patient’s condition at the time of transplantation. Auto-SCT resulted in better survival in patients who achieved CR after salvage therapy. By contrast, patients with PR or uncontrolled disease status may benefit from allo-SCT if they have an HLA-matched donor and a suitable condition for transplantation.

A recently published randomized phase 3 trial revealed no significant differences in survival between auto- and allo-SCT as a consolidation therapy for younger patients with poor-risk T-cell lymphomas. Schmitz N et al. suggested that standard chemotherapy followed by auto-SCT is a preferred option for younger patients [14]. Our results also demonstrated that auto-SCT should be considered as a consolidation therapy in transplant-eligible patients instead of allo-SCT. In particular, patients aged <50 years should actively consider transplantation, and auto-SCT can be performed based on the performance status even if the patient is aged ≥50 years. However, it remains unclear whether up-front SCT is mandatory in patients with T-cell lymphomas who showed good response to first-line chemotherapy. Despite the survival benefits of consolidative auto-SCT, the transplant frequency rate is only 10–20%, according to the COMPLETE study and US data [5, 15]. However, real-world data from the Swedish Lymphoma Registry revealed that patients who underwent up-front auto-SCT showed better long-term outcomes than those who did not undergo auto-SCT [16]. In the current study, the 3-year PFS and OS were 68.7% and 76.1% in patients with up-front SCT, respectively, with better survival in the auto-SCT group. Patients in good condition for transplantation were highly likely to be selected. Even so, upfront auto-SCT appears to have survival benefits considering the results of previous studies [3, 17, 18].

Most patients with relapsed/refractory T-cell lymphomas who did not undergo hematopoietic transplantation showed poor survival [19, 20]. Mak et al. reported that the median OS after relapse was only 5.5 months and 3-year OS and PFS after relapse were 18% and 11% in the absence of SCT [19]. The COMPLETE registry results revealed that SCT as a second-line treatment may improve survival in patients with the refractory disease [21]. In the current study, the estimated 3-year PFS and OS were 50.1% and 61.6%. Although we could not compare patients who only received salvage chemotherapy, our findings suggest that SCT may improve long-term survival in patients with relapsed/refractory disease. Furthermore, the selection of the type of transplantation depends on the patient’s disease status. According to our results, patients who achieved CR and underwent auto-SCT showed impressive long-term outcomes, with a 3-year OS of 79.5%. Although a small number of patients were examined in this study, the PR status before SCT also has advantages, showing improved PFS by allo-SCT and OS by auto-SCT. In the refractory group, the relapse rate was lower in patients who underwent allo-SCT than in those who underwent auto-SCT, but 50% of patients died in the allo-SCT group. In the relapsed group, patients who underwent allo-SCT showed better 3-year PFS and OS.

Previous studies have suggested that allo-SCT is an effective option for patients with relapsed/refractory disease [7, 10, 22]. However, lowering TRM risk is critical because most patients are older individuals who have been heavily pretreated [10, 23]. In the current study, most deaths occurred within a year of allo-SCT. Notably, in the subgroup analysis, patients who received the RIC regimen showed better survival than those who did not. Based on the patient’s transplant eligibility, allo-SCT with RIC may be a reliable curative option for patients with relapsed/refractory disease [24, 25]. In a retrospective analysis of the SFGM-TC registry, allo-SCT was performed as a salvage therapy in 147 patients with PTCL. Although there was no statistically significant difference in TRM between myeloablative conditioning and RIC (*p* = 0.09), Mamez et al. reported that the relapse rate was low even after treatment with RIC [22]. This suggests that the GVL effect is sufficient even in patients treated with RIC [22]. In an Italian study, Dodero et al. demonstrated that RIC is an effective therapeutic option for younger patients with chemosensitive diseases [10].

To compensate for the limitations of a retrospective study on patient assignment, we also performed survival analysis after applying propensity score matching. In patients with upfront transplantation, long-term outcomes were relatively similar to the entire patient group. In the salvage setting, the overall outcomes were not different. However, significant results could have been obtained if a larger number of patients were analyzed.

Due to certain limitations, the findings of the current study should be interpreted with caution. First, this was a retrospective study, and only patients enrolled in the KSBMT registry were analyzed. Second, due to the limitations of the KSBMT registry, clinical information of enrolled patients at the time of diagnosis was insufficient for analysis. Finally, only transplanted patients were analyzed, and patients with and without transplantation were not compared. Therefore, to identify the transplantation rate among all aggressive T-cell lymphomas and compare the survival benefits of SCT with chemotherapy alone, a study is currently being conducted using clinical data from T-cell lymphomas registered in National Health Insurance over the past 20 years.

In conclusion, consolidative auto-SCT demonstrated a survival benefit in patients with aggressive T-cell lymphomas who responded to chemotherapy. Patients who achieved CR after salvage therapy for relapsed/refractory diseases may also benefit from auto-SCT. If the disease persists or cannot be controlled, allo-SCT can be considered, and RIC may be an option to reduce TRM risk. To maximize the effect of transplantation, further research is warranted to determine which patients might benefit from auto- or allo-SCT.

## Supplementary information

Supplemental materials

## Data Availability

The datasets generated during and/or analyzed during the current study are available from the corresponding author on reasonable request.
