# Supplementary material for: Real-world data of long-term survival in patients with T-cell lymphoma who underwent stem cell transplantation
Source: Blood Cancer J. 2023 Jun 26;13(1):95. doi: 10.1038/s41408-023-00868-w (PMC10293168; doi:10.1038/s41408-023-00868-w)
Supplement: Supplementary file 1 — Supplemental materials [file 41408_2023_868_MOESM1_ESM.docx]

**Title:** Real-world data of long-term survival in patients with T-cell lymphoma who underwent stem cell transplantation

**Running title:** Outcomes of stem cell transplantation in T-cell lymphomas

**Authors:** Dong Won Baek, Joon Ho Moon, Jae Hoon Lee, Ka-Won Kang, Ho Sup Lee, Hyeon-Seok Eom, Enuyoung Lee, Ji Hyun Lee, Jeong-Ok Lee, Seong Kyu Park, Seok Jin Kim, Keon Hee Yoo, Sung-Soo Yoon, Youngil Koh, Hyoung Jin Kang, Jong-Ho Won, Chuhl Joo Lyu, Seung Min Hahn, Jung-Hee Lee, Joon Seong Park, Jae-Cheol Jo, Yeung-Chul Mun, Deok-Hwan Yang, Ga-Young Song, Sung-Nam Lim, Sang Kyun Sohn, and The Korean Society of Blood and Marrow Transplantation

**Supplementary Figure 1.** Survival outcomes. Progression-free survival (A) and overall survival (B) of all patients.

(A) PFS


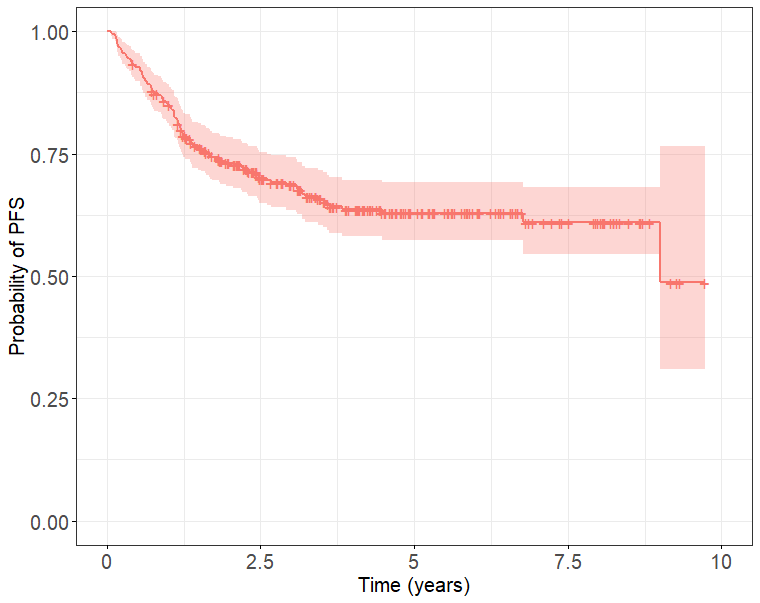


(B) OS


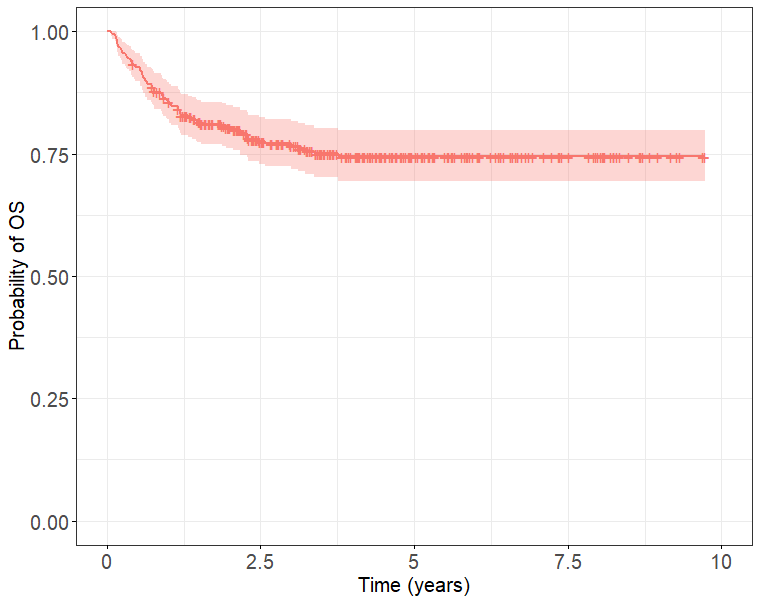


**Supplementary Figure 2.** Survival outcomes according to the first-line treatment response in patients who underwent up-front stem cell transplantation. Progression-free survival (A) and overall survival (B).

(A) PFS


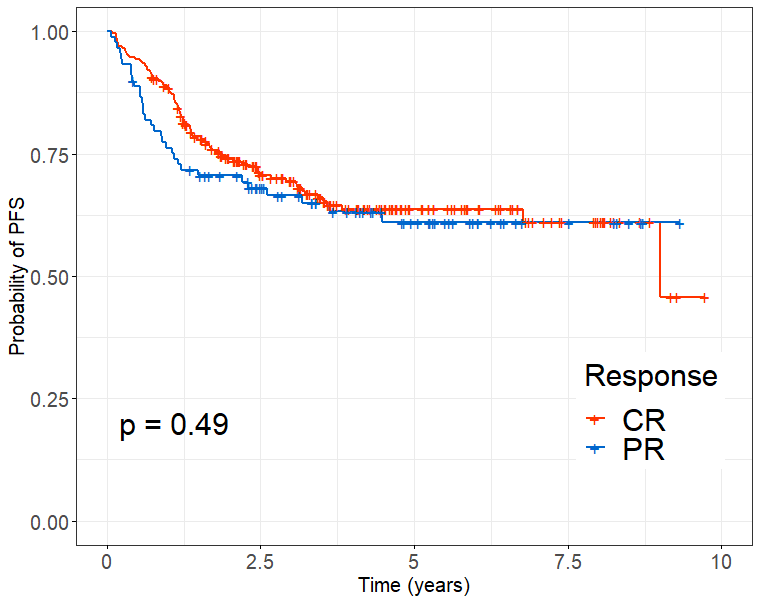


(B) OS


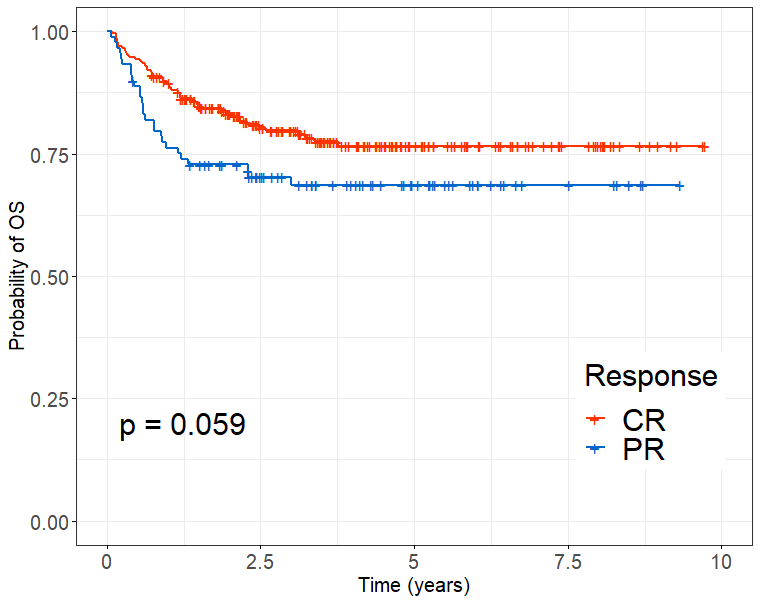


**Supplementary Figure 3.** Cumulative relapse incidence of the patients with CR (A), and PR (B), and non-relapse mortality of the patients with CR (C), and PR (D)

(A) Cumulative relapse incidence of CR patients (p-value = 0.23)


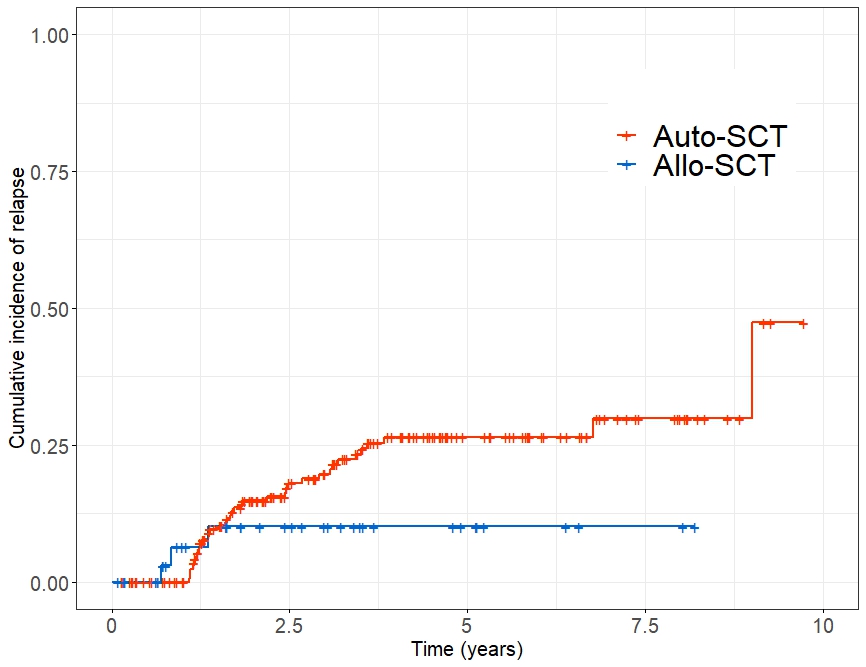


(B) Cumulative relapse incidence of PR patients (p-value = 0.86)


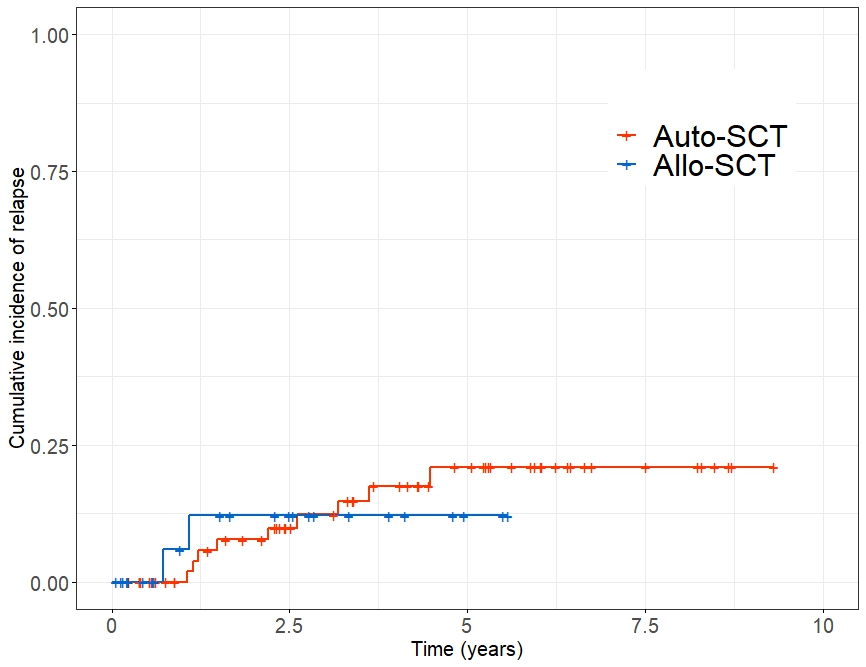


(C) Non-relapse mortality of CR patients (p-value = 0.054)


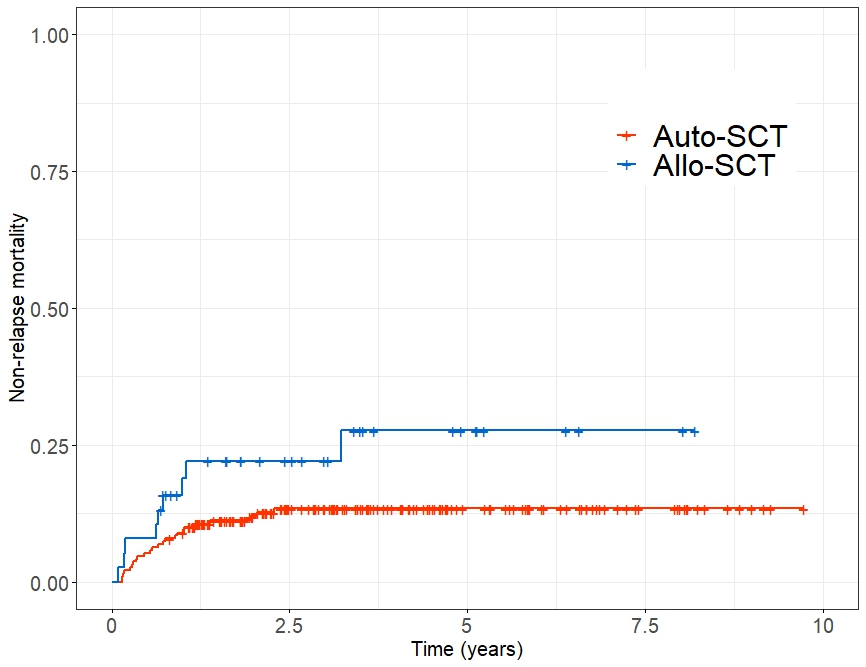


(D) Non-relapse mortality of PR patients (p-value = 0.049)


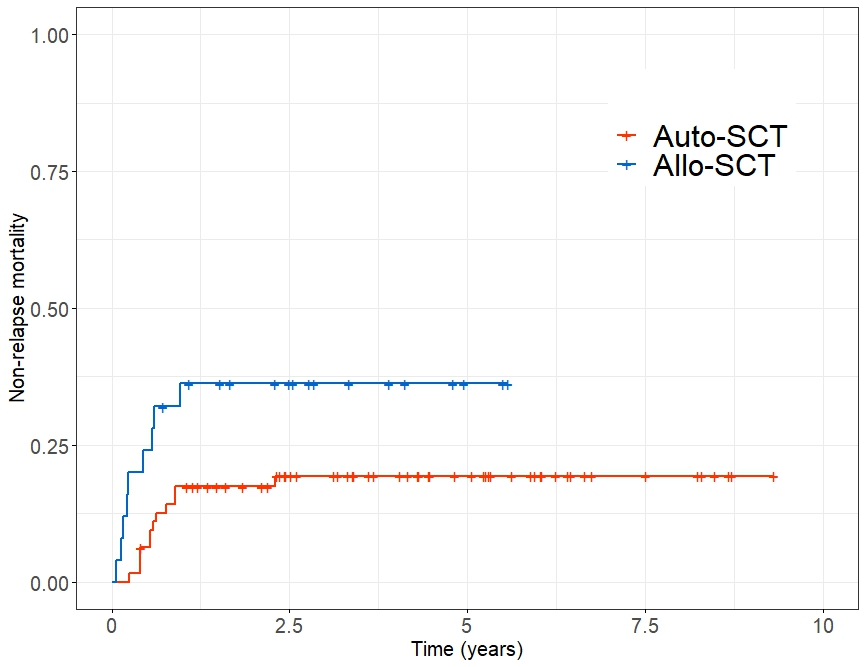


**Supplementary Figure 4.** Kaplan-Meier curve. Patients aged < 50 years exhibited better progression-free survival (PFS) (A) and overall survival (OS) (B) than older patients. There were no survival differences between auto- and allo-SCT in terms of PFS (C), and OS (D) in patients aged < 50 years. In older patients aged ≥ 50 years, patients who underwent auto-SCT showed better tendency of PFS (E), and improved OS (F) than those with allo-SCT.

(A) PFS


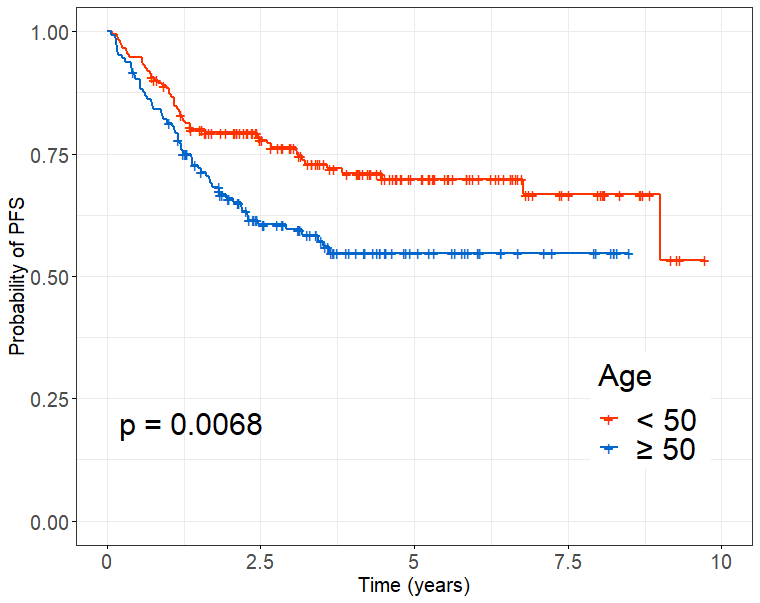


(B) OS


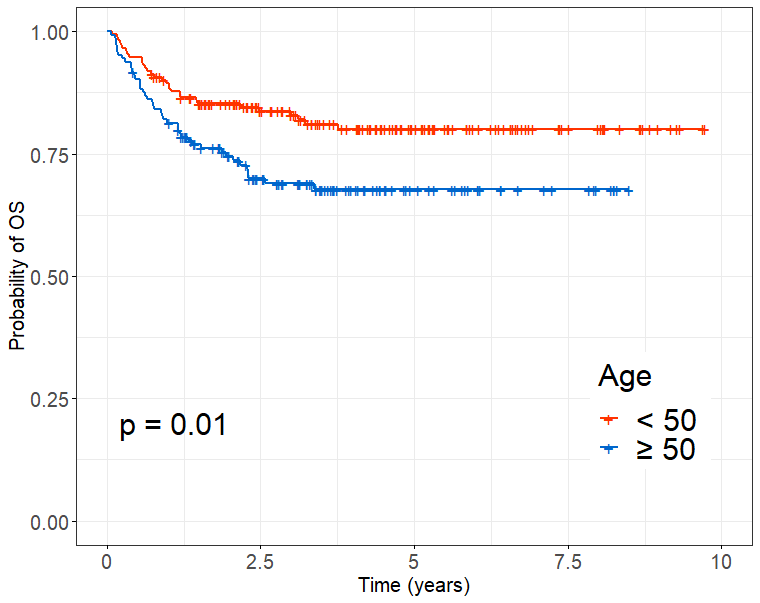


(C) PFS in patients aged < 50 years


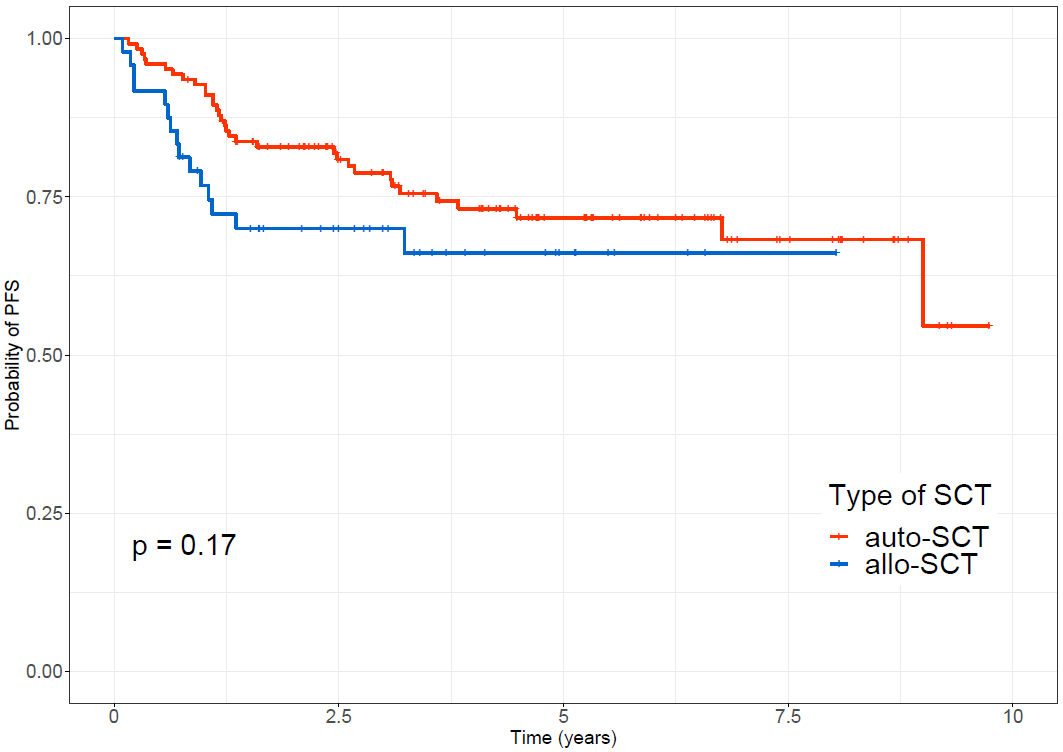


(D) OS in patients aged <5 0 years


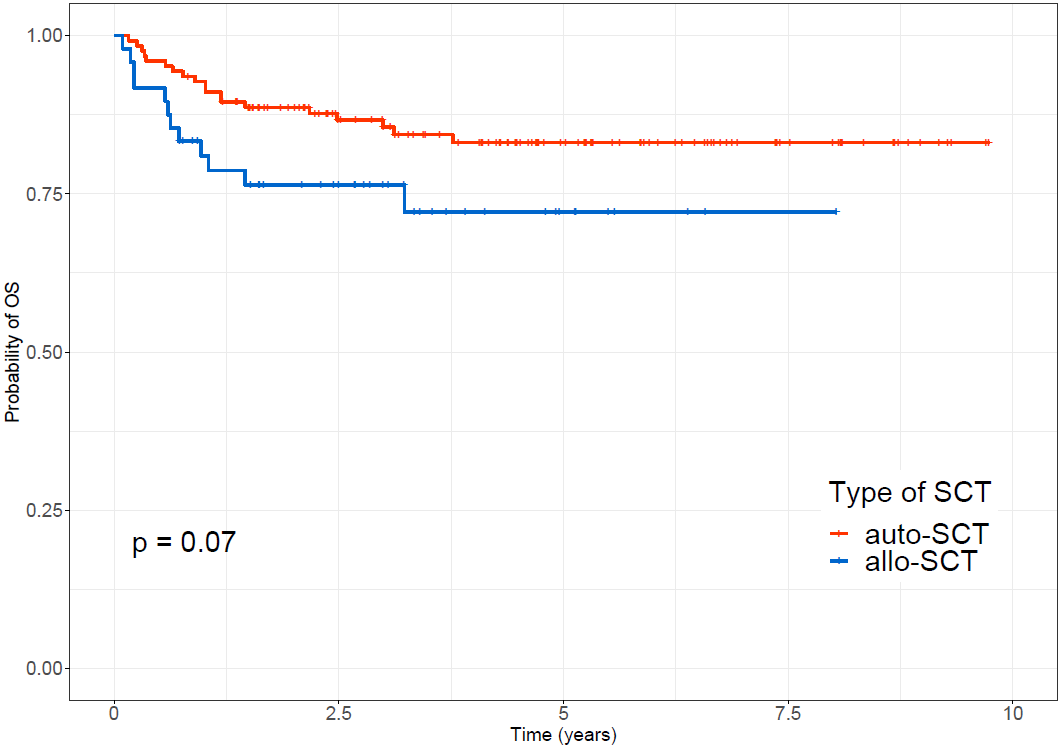


(E) PFS in patients aged ≥ 50 years


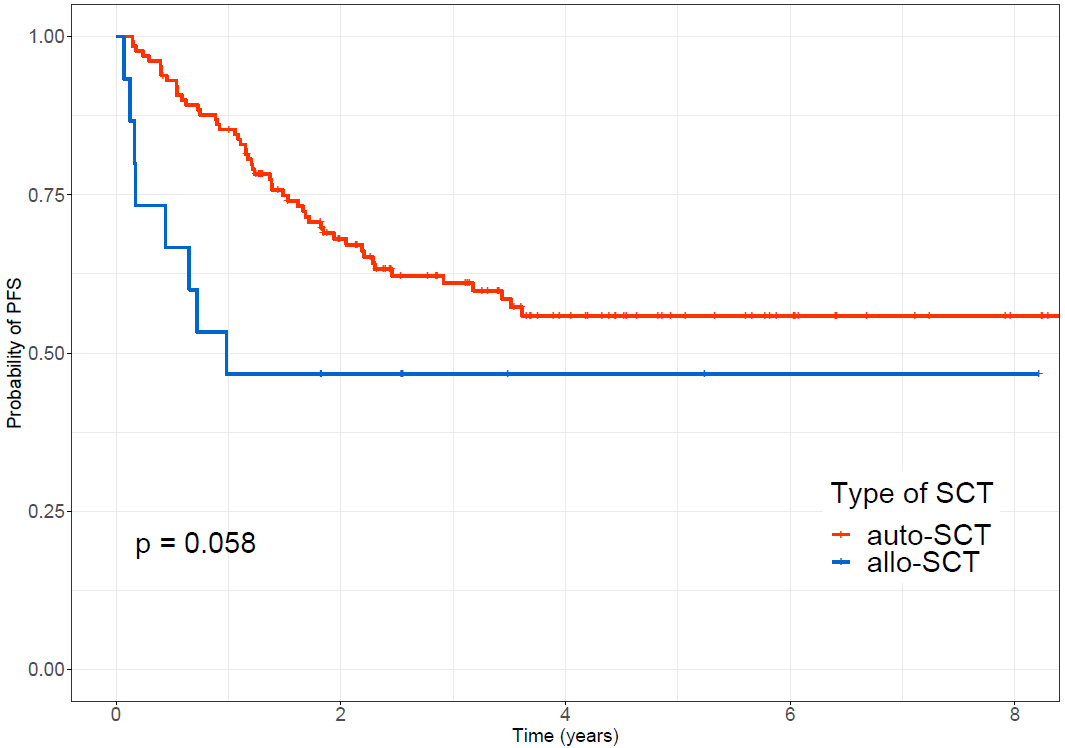


(F) OS in patients aged ≥ 50 years


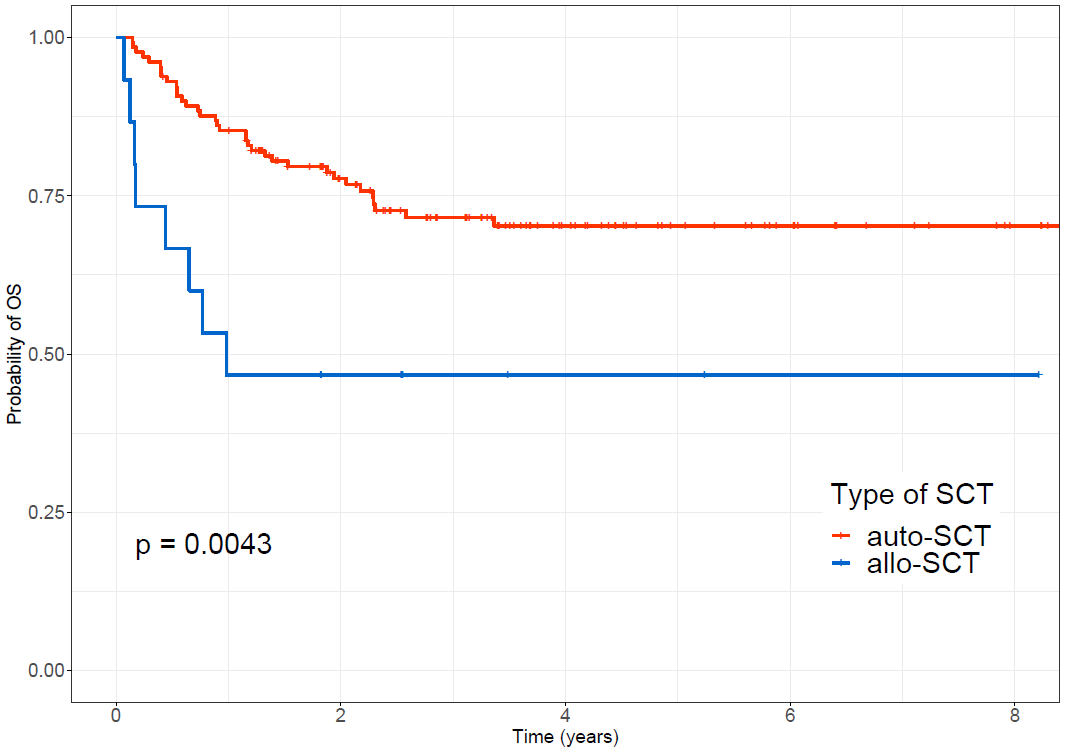


**Supplementary Figure 5.** Propensity score matched analysis in patients with up-front SCT. (A) Results of propensity score matching, (B) PFS in patients with CR/PR who received up-front SCT, (C) OS in patients with CR/PR who received up-front SCT.

(A) Propensity score matching results


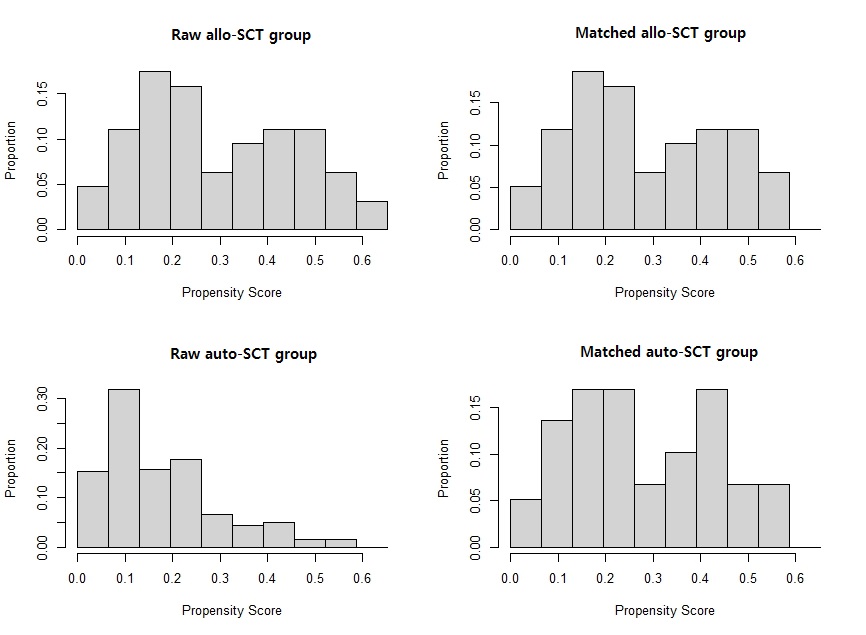


(B) PFS


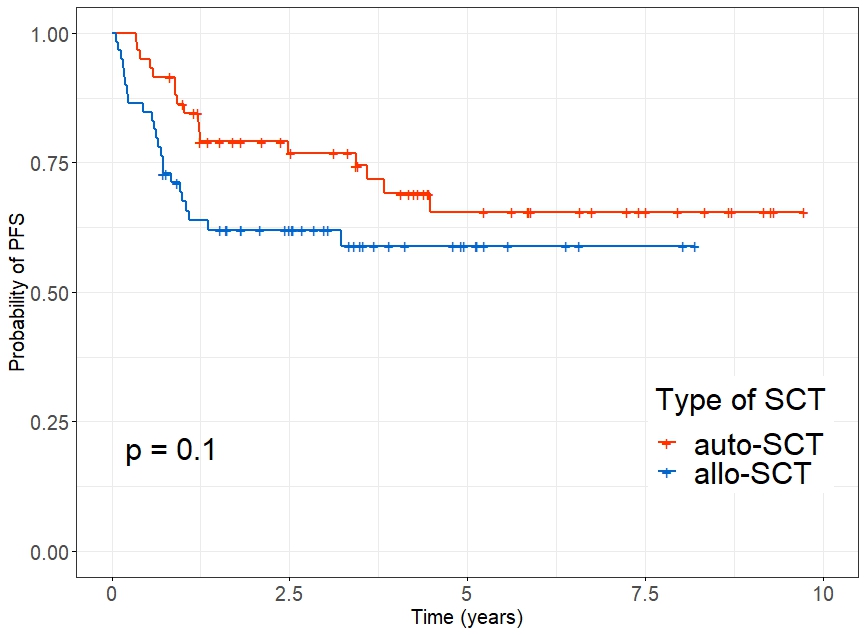


(C) OS


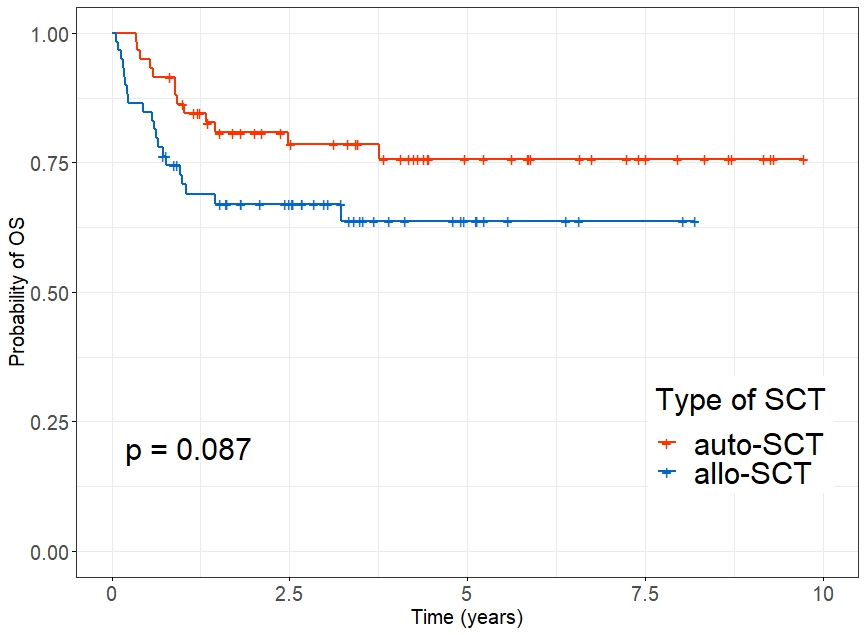


**Supplementary Figure 6.** Cumulative relapse incidence of the patients with CR/PR (A), and relapsed/refractory status (B), and non-relapse mortality of the patients with CR/PR (C), and relapsed/refractory status (D)

(A) Cumulative relapse incidence of CR/PR patients (p-value = 0.005)


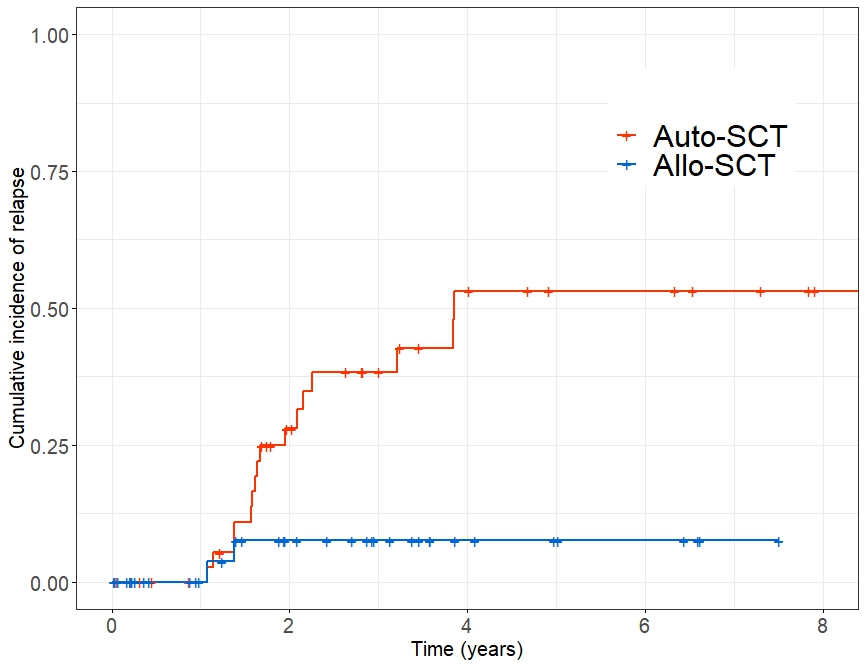


(B) Cumulative relapse incidence of relapsed/refractory status patients (p-value = 0.13)


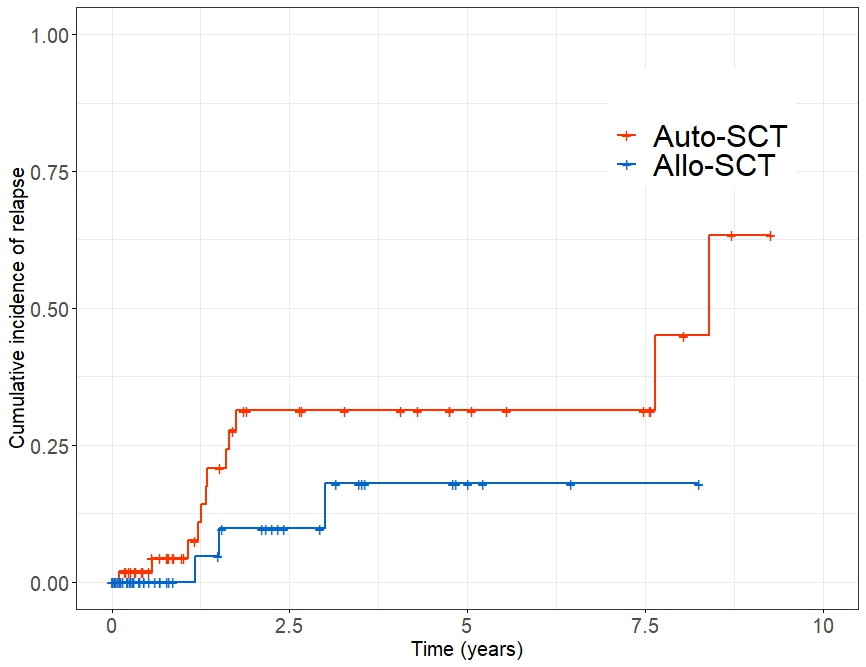


(C) Non-relapse mortality of CR/PR patients (p-value < 0.001)


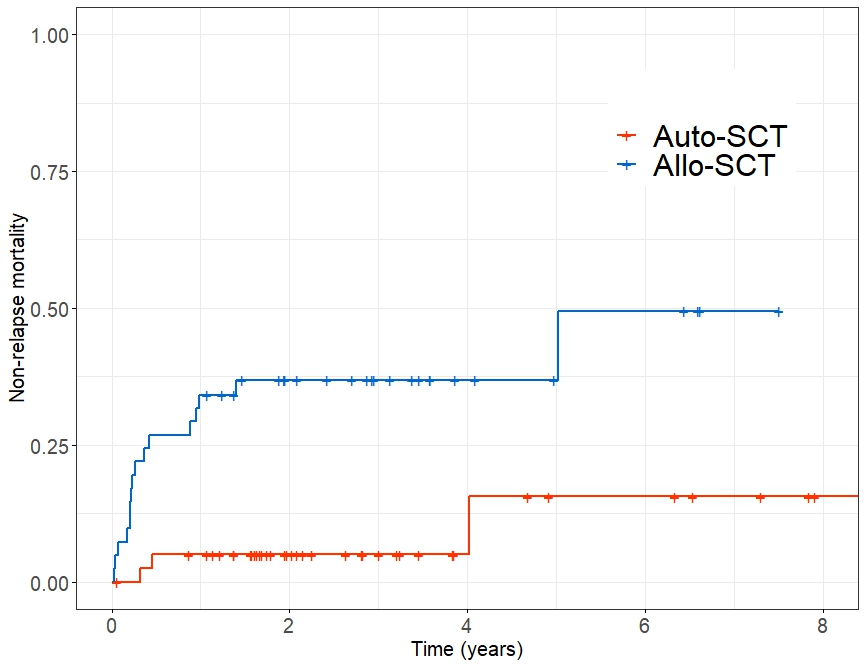


(D) Non-relapse mortality of relapsed/refractory status patients (p-value = 0.023)


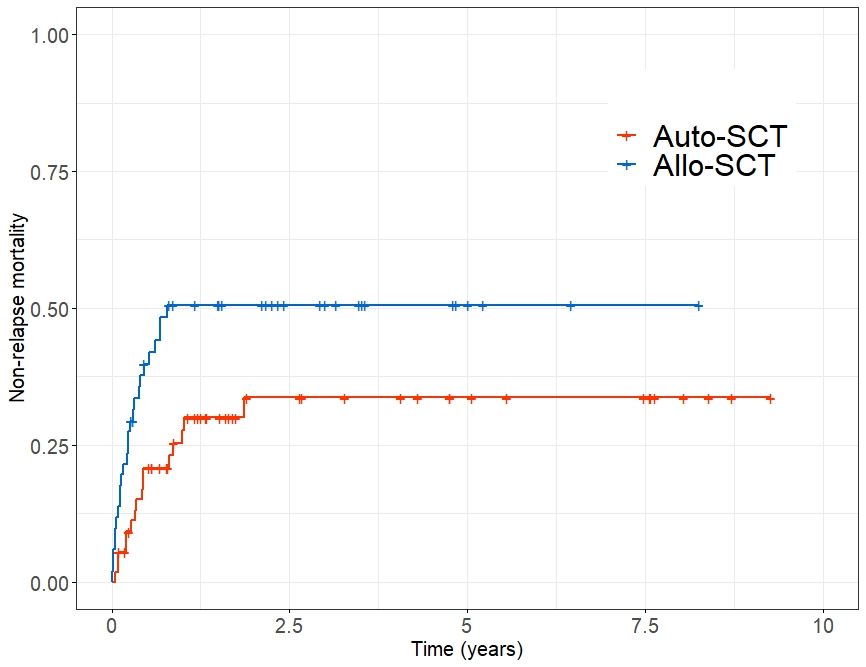


**Supplementary Figure 7.** Propensity score matched analysis in patients with salvage setting. (A) Results of propensity score matching in patients with CR/PR, (B) PFS in patients with CR/PR, (C) OS in patients with CR/PR, (D) Results of propensity score matching in patients with relapsed/refractory disease, (E) PFS in patients with relapsed/refractory disease, (F) OS in patients with relapsed/refractory disease.

(A) Propensity score matching results


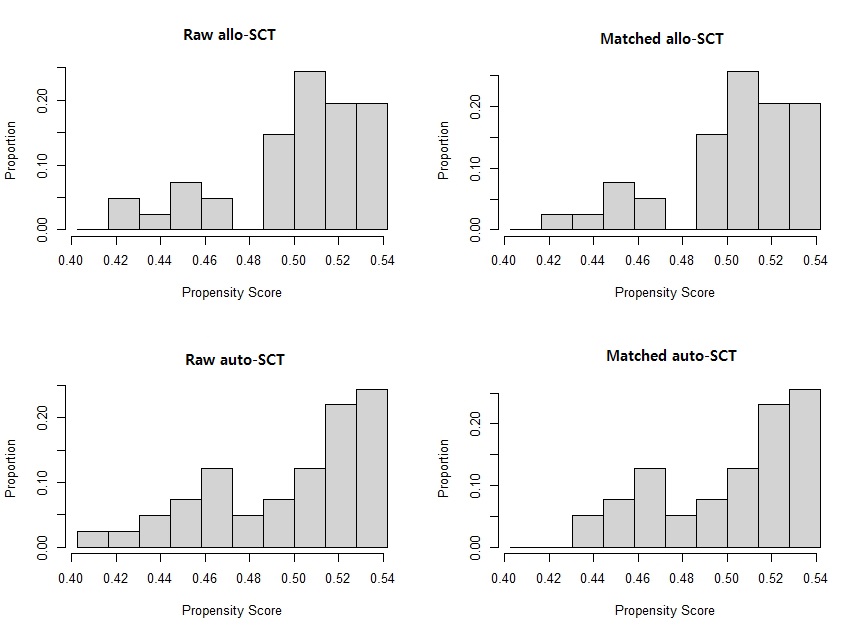


(B) PFS in patients with CR/PR


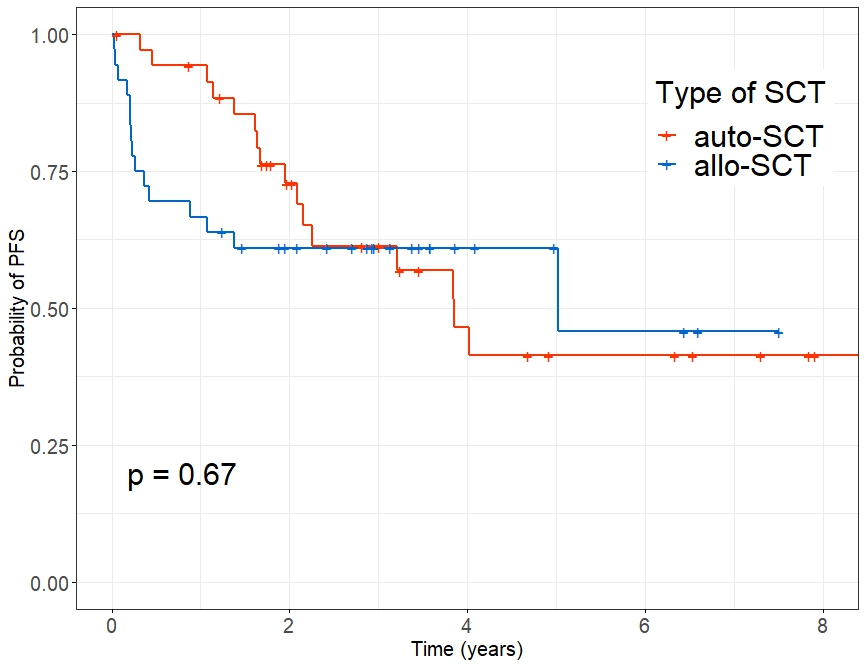


(C) OS in patients with CR/PR


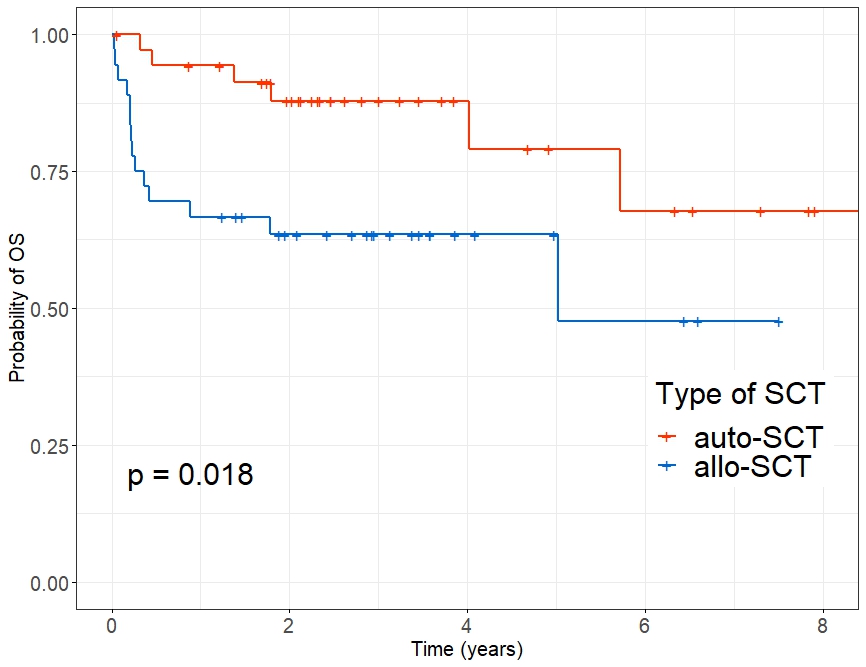


(D) Results of propensity score matching in patients with relapsed/refractory disease


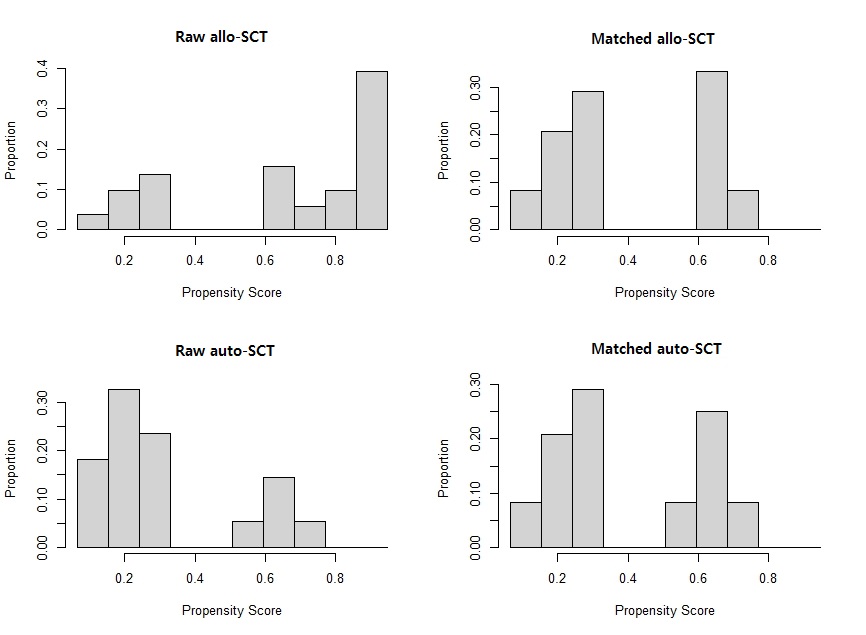


(E) PFS in patients with relapsed/refractory disease


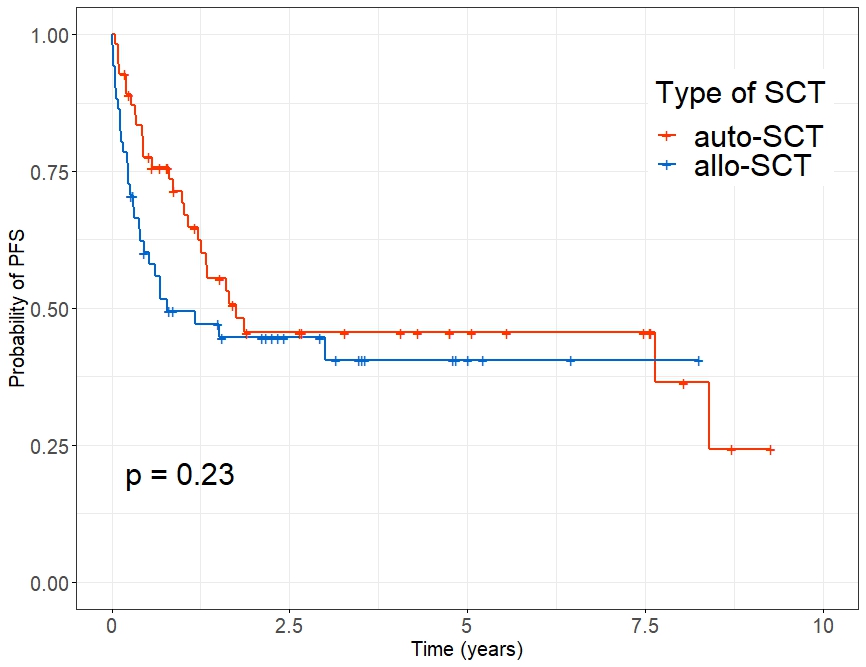


(F) OS in patients with relapsed/refractory disease


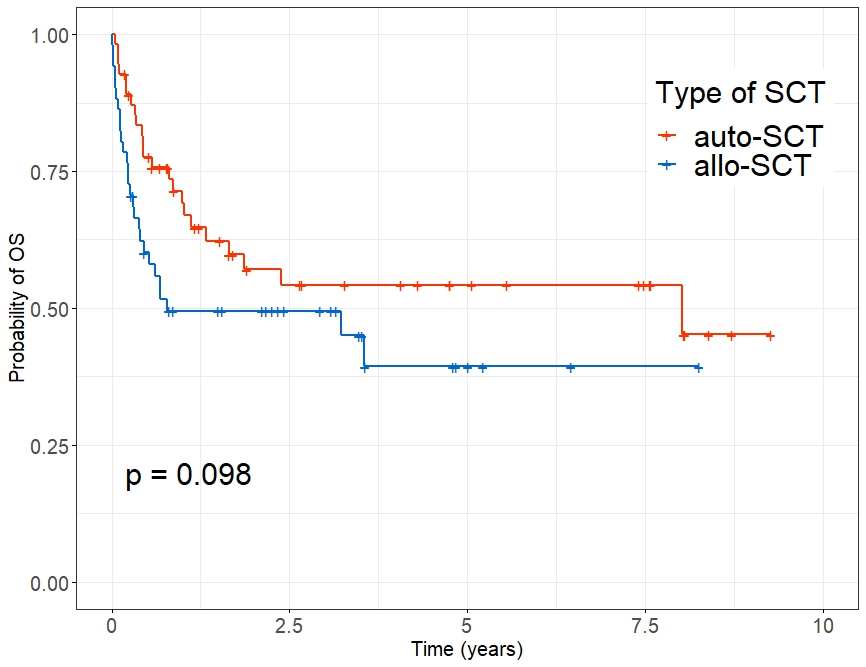


**Supplementary Tables**

**Supplementary Table 1.** Outcomes according to the disease status and transplantation type of patients who underwent up-front stem cell transplantation

| Response | SCT type | Relapse, n (%) | Death, n (%) | Death 100-days after SCT, n (%) | Death 1-yr after SCT, n (%) | 3-yr PFS, % (95% CI) | 3-yr OS, % (95% CI) | 5-yr PFS, % (95% CI) | 5-yr OS, % (95% CI) |
| --- | --- | --- | --- | --- | --- | --- | --- | --- | --- |
| CR  (n = 228) | Auto-SCT (n = 190) | 39/190 (20.5%) | 37/190 (19.5%) | 5/190 (2.6%) | 16/190 (8.4%) | 69.5% (0.63-0.77) | 80.7% (0.75-0.87) | 63.7% (0.57-0.72) | 77.9% (0.72-0.85) |
|  | Allo-SCT (n=38) | 3/38 (7.9%) | 10/38 (26.3%) | 3/38 (7.8%) | 7/38 (18.4%) | 70.0% (0.57-0.87) | 75.2% (0.62-0.91) | 65.0% (0.50-0.84) | 69.8% (0.55-0.89) |
| PR  (n = 89) | Auto-SCT (n = 64) | 9/64 (14.1%) | 17/64 (26.6%) | 1/64 (1.6%) | 11/64 (17.2%) | 70.8% (0.60-0.83) | 72.3% (0.62-0.85) | 63.8% (0.52-0.78) | - |
|  | Allo-SCT (n = 25) | 2/25 (8.0%) | 10/25 (40.0%) | 5/25 (20%) | 10/25 (40.0%) | 56.0% (0.40-0.79) | 60.0% (0.44-0.83) | - | - |

Allo-, allogeneic; Auto, autologous; CR, complete remission; OS, overall survival; PFS, progression-free survival; PR, partial remission; SCT: stem cell transplantation; yr, year

**Supplementary Table 2**. Causes of death in patients who received up-front stem cell transplantation (n=74).

| Auto-SCT (n=54) | No. (%) |
| --- | --- |
| Infection | 20 (37.0) |
| Disease progression | 18 (33.3) |
| Cardiac cause | 2 (3.7) |
| Secondary malignancy | 1 (1.9) |
| Hepatitis | 1 (1.9) |
| Hemorrhage | 1 (1.9) |
| Others | 11 (20.4) |
| Allo-SCT (n=20) | No. (%) |
| Infection | 11 (55.0) |
| Disease progression | 2 (10.0) |
| GVHD | 4 (20.0) |
| Others | 3 (15.0) |

Allo-SCT, allogeneic stem cell transplantation; auto-SCT, autologous stem cell transplantation; GVHD, graft versus host disease

**Supplementary Table 3.** Factors affecting long-term survival in patients who underwent up-front stem cell transplantation

1) Progression-free survival

|  | Univariate | | |  | Multivariate | | |
| --- | --- | --- | --- | --- | --- | --- | --- |
|  | HR | 95% CI | p-value |  | HR | 95% CI | p-value |
| Age at SCT, ≥ 50 vs. < 50 | 1.89 | 1.267-2.825 | 0.002 |  | 1.88 | 1.260-2.802 | 0.002 |
| Sex, female vs. male | 0.84 | 0.551-1.287 | 0.427 |  |  |  |  |
| Disease status at SCT, PR vs. CR | 1.13 | 0.743-1.710 | 0.573 |  |  |  |  |
| SCT type, allo-SCT vs. auto-SCT | 1.68 | 1.038-2.732 | 0.035 |  | 1.70 | 1.053-2.758 | 0.030 |

2) Overall survival

|  | Univariate | | |  | Multivariate | | |
| --- | --- | --- | --- | --- | --- | --- | --- |
|  | HR | 95% CI | p-value |  | HR | 95% CI | p-value |
| Age at SCT, ≥ 50 vs. < 50 | 2.24 | 1.368-3.651 | 0.001 |  | 2.23 | 1.366-3.644 | 0.001 |
| Sex, female vs. male | 0.90 | 0.540-1.491 | 0.677 |  |  |  |  |
| Disease status at SCT, PR vs. CR | 1.50 | 0.933-2.424 | 0.094 |  | 1.52 | 0.943-2.441 | 0.086 |
| SCT type, allo-SCT vs. auto-SCT | 2.23 | 1.293-3.861 | 0.004 |  | 2.24 | 1.298-3.869 | 0.004 |

Allo-, allogeneic; Auto, autologous; CI, confidence interval; CR, complete remission; HR, hazard ratio; PR, partial remission; SCT: stem cell transplantation

**Supplementary Table 4.** Outcomes according to the disease status and transplantation type of patients who underwent stem cell transplantation as a part of salvage therapy

| Disease status at the time of SCT | Type of SCT | Progression, n (%) | Death, n (%) | Death within 100-days after SCT, n (%) | Death within 1-yr after SCT, n (%) | 3-yr PFS, % (95% CI) | 3-yr OS, % (95% CI) |
| --- | --- | --- | --- | --- | --- | --- | --- |
| CR (n = 64) | Auto-SCT (n = 32) | 13 (40.6%) | 4 (12.5%) | 0 | 1 (7.7%) | 60.3% (0.44-0.82) | 79.5% (0.57-1.00) |
|  | Allo-SCT (n = 33) | 2 (6.1%) | 14 (42.4%) | 7 (21.2%) | 11 (33.3%) | 57.6% (0.43-0.77) | 60.3% (0.46-0.80) |
| PR (n = 17) | Auto-SCT (n = 9) | 3 (33.3%) | 3 (33.3%) | 0 | 1 (11.1%) | 53.3% (0.28-1.00) | 66.7% (0.42-1.00) |
|  | Allo-SCT (n = 8) | 0 | 3 (37.5%) | 2 (25.0%) | 3 (37.5%) | 62.5% (0.37-1.00) | 62.5% (0.37-1.00) |
| Refractory (n = 65) | Auto-SCT (n = 47) | 10 (21.3%) | 19 (40.4%) | 4 (8.5%) | 11 (23.4%) | 32.9% (0.14-0.78) | 47.6% (0.29-0.79) |
|  | Allo-SCT (n = 18) | 1 (5.6%) | 10 (55.6%) | 7 (38.9%) | 9 (50.0%) | 35.6% (0.17-0.76) | 35.6% (0.17-0.76) |
| Relapsed (n = 41) | Auto-SCT (n = 8) | 2 (25.0%) | 6 (75.0%) | 2 (25.0%) | 5 (62.5%) | 25.0% (0.75-083) | 37.5% (0.15-0.92) |
|  | Allo-SCT (n = 33) | 2 (6.1%) | 17 (51.5%) | 8 (24.2%) | 16 (48.5%) | 43.3% (0.29-0.65) | 50.5% (0.36-0.71) |

Allo-, allogeneic; Auto, autologous; CR, complete remission; OS, overall survival; PFS, progression-free survival; PR, partial remission; SCT: stem cell transplantation; yr, year

**Supplementary Table 5**. Causes of death in patients who received stem cell transplantation in a salvage setting (n=76)

| CR/PR  (n=24) | Auto-SCT (n=7) | No. (%) |
| --- | --- | --- |
|  | Disease progression | 4 (57.1) |
|  | Infection | 1 (14.3) |
|  | Others | 2 (28.6) |
|  | Allo-SCT (n=17) | No. (%) |
|  | Infection | 10 (58.8) |
|  | GVHD | 2 (11.8) |
|  | Disease progression | 1 (5.9) |
|  | Hepatitis | 1 (5.9) |
|  | Cardiac cause | 1 (5.9) |
|  | Others | 2 (11.8) |
| Relapsed/Refractory  (n=52) | Auto-SCT (25) | No. (%) |
|  | Infection | 11 (44.0) |
|  | Disease progression | 9 (36.0) |
|  | Cardiac cause | 1 (4.0) |
|  | Others | 4 (16.0) |
|  | Allo-SCT (27) | No. (%) |
|  | Infection | 17 (63.0) |
|  | GVHD | 5 (18.5) |
|  | Disease progression | 2 (7.4) |
|  | Cardiac cause | 2 (7.4) |
|  | Hemorrhage | 1 (3.7) |

Allo-SCT, allogeneic stem cell transplantation; auto-SCT, autologous stem cell transplantation; CR, complete remission; GVHD, graft versus host disease, PR, partial remission

**Supplementary Table 6.** Factors affecting long-term survival in patients with CR and PR who underwent stem cell transplantation as a salvage therapy

1) Progression-free survival

|  | Univariate | | |  | Multivariate | | |
| --- | --- | --- | --- | --- | --- | --- | --- |
|  | HR | 95% CI | p-value |  | HR | 95% CI | p-value |
| Age at SCT, ≥ 50 vs. < 50 | 1.75 | 0.887-3.449 | 0.107 |  |  |  |  |
| Female vs. Male | 0.61 | 0.264-1.424 | 0.255 |  |  |  |  |
| Previous SCT, No vs. Yes | 1.92 | 0.907-4.079 | 0.187 |  |  |  |  |
| Allogeneic-SCT vs. Autologous-SCT | 0.80 | 0.382-1.685 | 0.561 |  |  |  |  |
| CR vs. PR | 0.86 | 0.371-1.994 | 0.725 |  |  |  |  |

2) Overall survival

|  | Univariate | | |  | Multivariate | | |
| --- | --- | --- | --- | --- | --- | --- | --- |
|  | HR | 95% CI | p-value |  | HR | 95% CI | p-value |
| Age at SCT, ≥ 50 vs. < 50 | 1.10 | 0.450-2.685 | 0.835 |  |  |  |  |
| Female vs. Male | 0.43 | 0.124-1.471 | 0.178 |  |  |  |  |
| Previous SCT, No vs. Yes | 1,09 | 0.433-2.760 | 0.851 |  |  |  |  |
| Allogeneic-SCT vs. Autologous-SCT | 2.81 | 1.023-7.694 | 0.045 |  | 3.03 | 1.256-7.327 | 0.014 |
| CR vs. PR | 1.66 | 0.599-4.584 | 0.330 |  |  |  |  |

CI, confidence interval; CR, complete remission; HR, hazard ratio; PR, partial remission; SCT: stem cell transplantation

**Supplementary Table 7.** Factors affecting long-term survival in patients with relapsed/refractory status who underwent stem cell transplantation as a salvage therapy

1) Progression-free survival

|  | Univariate | | |  | Multivariate | | |
| --- | --- | --- | --- | --- | --- | --- | --- |
|  | HR | 95% CI | p-value |  | HR | 95% CI | p-value |
| Age at SCT, ≥ 50 vs. < 50 | 0.96 | 0.553-1.662 | 0.881 |  |  |  |  |
| Female vs. Male | 1.03 | 0.568-1.855 | 0.932 |  |  |  |  |
| Previous SCT, No vs. Yes | 0.27 | 0.125-0.589 | <0.001 |  | 0.27 | 0.126-0.581 | <0.001 |
| Allogeneic-SCT vs. Autologous-SCT | 0.87 | 0.451-1.668 | 0.670 |  |  |  |  |
| Relapse vs. Refractory | 0.45 | 0.226-0.901 | 0.024 |  | 0.44 | 0.229-0.855 | 0.015 |

2) Overall survival

|  | Univariate | | |  | Multivariate | | |
| --- | --- | --- | --- | --- | --- | --- | --- |
|  | HR | 95% CI | p-value |  | HR | 95% CI | p-value |
| Age at SCT, ≥ 50 vs. < 50 | 1.05 | 0.585-1.897 | 0.863 |  |  |  |  |
| Female vs. Male | 1.03 | 0.550-1.022 | 0.932 |  |  |  |  |
| Previous SCT, No vs. Yes | 0.27 | 0.123-0.612 | 0.002 |  | 0.28 | 0.126-0.610 | 0.001 |
| Allogeneic-SCT vs. Autologous-SCT | 0.97 | 0.378-1.742 | 0.525 |  |  |  |  |
| Relapse vs. Refractory | 0.49 | 0.238-1.014 | 0.055 |  | 0.50 | 0.251-0.986 | 0.045 |

CI, confidence interval; CR, complete remission; HR, hazard ratio; PR, partial remission; SCT: stem cell transplantation

**Supplementary Table 8.** Transplantation features of patients who underwent allo-SCT as a part of salvage therapy according to the conditioning intensity (n = 92).

| Characteristics | RIC | MAC | p-value |
| --- | --- | --- | --- |
|  | No. (%) | No. (%) |  |
| No. of patients | 63 (68.5) | 29 (31.5) |  |
| Age at the time of SCT, years |  |  | 0.326 |
| Median (range) | 44.0 (15.1-70.9) | 40.6 (15.3-63.4) |  |
| ≤ 20 | 5 (7.9) | 8 (27.6) |  |
| 21 - 30 | 9 (14.3) | 2 (6.9) |  |
| 31 - 40 | 6 (9.5) | 5 (17.2) |  |
| 41 - 50 | 13 (20.6) | 5 (17.2) |  |
| 51 - 60 | 23 (36.5) | 7 (24.1) |  |
| > 60 | 7 (11.1) | 2 (6.9) |  |
| Sex |  |  | 0.108 |
| Male | 43 (68.3) | 25 (86.2) |  |
| Female | 20 (31.7) | 4 (13.8) |  |
| Histopathology |  |  | 0.185 |
| PTCL-NOS | 25 (39.7) | 14 (48.3) |  |
| Extranodal NK/T-cell lymphoma | 9 (14.3) | 6 (20.7) |  |
| AITL | 15 (23.8) | 5 (17.2) |  |
| ALCL | 11 (17.5) | 4 (13.8) |  |
| EATL | 2 (3.2) | 0 |  |
| Others | 1 (1.6) | 0 |  |
| Disease status at the time of SCT |  |  | 0.665 |
| CR2 | 19 (30.2) | 11 (37.9) |  |
| > CR2 | 3 (4.8) | 0 |  |
| ≥ PR2 | 6 (9.5) | 2 (6.9) |  |
| Refractory | 13 (20.6) | 5 (17.2) |  |
| Relapsed | 22 (34.9) | 11 (37.9) |  |
| No. of SCT |  |  | 0.148 |
| 1 | 54 (85.7) | 21 (72.4) |  |
| 2 | 7 (11.1) | 8 (27.6) |  |
| ≥ 3 | 2 (3.2) | 0 |  |
| Graft source |  |  | 1.000 |
| Peripheral blood stem cell | 61 (96.8) | 28 (96.6) |  |
| Bone marrow | 1 (1.6) | 0 |  |
| Cord blood | 1 (1.6) | 1 (3.4) |  |
| HLA matching |  |  | 0.291 |
| Sibling identical | 23 (36.5) | 11 (37.9) |  |
| Matched unrelated donor | 22 (34.9) | 15 (51.7) |  |
| Mismatched unrelated donor | 0 | 1 (3.4) |  |
| Haploidentical | 17 (27.0) | 1 (3.4) |  |
| Cord blood | 1 (1.6) | 1 (3.4) |  |
| Total body irradiation |  |  | 0.990 |
| Performed | 24 (38.7%) | 12 (41.4%) |  |
| T depletion |  |  | 0.899 |
| ATG | 58 (92.0) | 28 (96.6) |  |
| Infused CD34+ cells |  |  | 0.824 |
| Median (range) | 5.95 (1.08-17.7) | 5.34 (1.4-17.9) |  |

AITL, angioimmunoblastic T-cell lymphoma; ALCL, anaplastic large-cell lymphoma; ALK, anaplastic lymphoma kinase; CR, complete remission; EATL, enteropathy-associated T-cell lymphoma; MAC, myeloablative conditioning; PR, partial remission; PTCL-NOS, peripheral T-cell lymphoma not otherwise specified; RIC, reduced intensity conditioning; SCT, stem cell transplantation
